# Enhanced Flame Retardancy of Styrene-Acrylic Emulsion Based Damping Composites Based on an APP/EG Flame-Retardant System

**DOI:** 10.3390/ma16113894

**Published:** 2023-05-23

**Authors:** Jingxing Wu, Jianhua Bi, Baoluo Xu, Lisha Fu, Wanjun Hao

**Affiliations:** State Key Laboratory of Marine Resources Utilization in the South China Sea, School of Materials Science and Engineering, Hainan University, Haikou 570100, China; 20080500210032@1hainanu.edu.cn (J.W.);

**Keywords:** ammonium polyphosphate (APP), expandable graphite (EG), flame retardancy, damping performance

## Abstract

Developing flame-retarded styrene-acrylic emulsion (SAE) based damping composites is a challenging task because of their very high flammability. A promising approach is the synergistic combination of expandable graphite (EG) and ammonium polyphosphate (APP). In this study, the surface modification of APP was modified by commercial titanate coupling agent ndz-201 through ball milling, and the SAE-based composite material was prepared with SAE and different ratios of modified ammonium polyphosphate (MAPP) and EG. The surface of MAPP was successfully chemically modified by NDZ-201 through scanning electron microscopy (SEM), Fourier transform infrared spectroscopy (FTIR), X-ray diffraction analysis (XRD), Energy Dispersion Spectroscopy (EDS), and contact angle. The effects of different ratios of MAPP and EG on the dynamic and static mechanical properties and flame retardancy of composite materials were explored. The results showed that when MAPP:EG = 1:4, the limiting oxygen index (LOI) of the composite material was 52.5%, and the vertical burning test (UL-94) was at the V0 level. Its LOI increased by 141.9% compared to the composite materials without flame retardant. The optimized formulation of MAPP and EG in SAE-based damping composite materials showed a significant synergistic effect on the flame retardancy of the composite material.

## 1. Introduction

Styrene-acrylic emulsion (SAE) is used as a damping coating for train cars, ship interiors, and buildings because of its low volatile organic compounds (VOCs), good weather resistance, excellent viscoelastic properties, and strong adhesion to metal substrates. However, as the main component of damping composite materials, styrene-acrylic emulsion is greatly limited in practical applications due to its disadvantages of low limiting oxygen index and easy ignition. Therefore, in order to promote the wider application of styrene-acrylic materials, finding a suitable method to enhance their flame-retardant properties is an urgent problem that needs to be solved.

The damping properties of a polymer are dominated by its glass transition temperature where the chain segments in a polymer backbone make de Gennes reptation motions, molecular vibrational energy is converted into heat energy, and a loss peak appears in a certain temperature range in dynamic mechanical analysis measurement curves [1]. At a given frequency, adding specific fillers to the polymer can improve the damping performance of the polymer by affecting the glass transition, such as wollastonite [2], graphite oxide [3], and mica [4]. The layered structure of mica can cause interfacial friction with resin to increase energy loss, and its addition to polymers can play an excellent damping role. In application, the fire resistance of polymer needs to be considered. Although mica, as a clay mineral, can reduce the heat release rate [5], when considering parameters such as LOI and UL-94, adding mica alone cannot provide satisfactory fire resistance [6,7].

At a specific stage of the material combustion process, such as during heating, decomposition, ignition, or flame diffusion, flame retardants can interfere with combustion. Intumescent flame retardants are considered environmentally friendly due to their low toxicity, low smoke, and halogen-free properties. The addition of intumescent flame retardant (IFR) to the polymer matrix has the advantages of being a simple process and providing a significant improvement in flame retardancy [8,9,10,11,12,13,14]. Ammonium polyphosphate (APP) is a type of phosphorus nitrogen flame retardant, which can act as both an acid source and a gas source to provide a synergistic effect to form an expanded protective burnt layer, which serves as a physical barrier to isolate the underlying polymer from heat and flame [15]. When ammonium polyphosphate is applied in polymers, its high loading capacity and weak polymer compatibility can reduce the mechanical strength of the polymer [16]. Surface modification through coupling agents [17,18,19], microencapsulation with shell materials [20], and other methods can enhance the compatibility between APP and polymer matrix. However, after microencapsulation modification of the APP, substances may cause harm to the human body, such as the free formaldehyde after microencapsulation with melamine formaldehyde resin [21]. Compared to using coupling agents to treat the APP, it appears to be more green and environmentally friendly.

Some researchers added APP and other flame retardants jointly to the material to enhance the flame-retardant effect of the compound flame retardant via the synergistic effect of the compound flame retardant [22,23,24,25,26,27,28,29]. Expandable graphite (EG) is used as a flame retardant and forms carbon foam on its own. However, the flame-retardant ability is not enough when used alone, and the phenomenon of “popcorn” exists, which cannot suppress smoke and prevent molten droplet generation. Using EG and APP together to form an APP-EG flame-retardant system is an efficient way to improve the flame retardancy of polymers [30,31,32,33,34,35,36]. Li et al. [37] studied synergistic flame-retardant polyurethane foam materials with different particle sizes of EG and APP and found that the larger the particle size of EG, the more obvious the synergistic effect of EG and APP. Yao et al. [38] added EG and APP as flame retardants to polyurethane foam (SPUF), and the ultimate oxygen index of the composites increased by 51% compared with SPUF at a total amount of 20% and 2:1 APP:EG. Guo et al. [39] reported the addition of APP and EG as synergistic flame retardants to jute/polypropylene composites, and the results showed that the combination of APP and EG greatly improved the flame-retardant properties and mechanical properties of the composites. Although APP-EG systems are currently used in many polymeric materials, they are rarely seen in styrene-acrylic emulsion damping composite materials.

The purpose of this study was to improve the flame retardancy of SAE composite materials as much as possible without sacrificing mechanical properties. We innovatively used a ball milling process and low-cost titanate coupling agent NDZ-201 to modify APP to enhance the compatibility of ammonium polyphosphate and polymer. We then added modified ammonium polyphosphate (MAPP) and expandable graphite to SAE damping emulsion in different ratios to study the flame-retardant properties, damping properties, and mechanical properties of damping composite materials with different ratios of APP and EG. MAPP was tested using scanning electron microscopy (SEM), Fourier transform infrared spectroscopy (FTIR), X-ray diffraction analysis (XRD), Energy Dispersion Spectroscopy (EDS), and the contact angle; results demonstrated the successful preparation of MAPP. The flammability of the composite was evaluated using LOI and UL-94 tests, and the microscopic action mechanism of the composite flame retardant on the damping material was analyzed. In addition, the dynamic mechanical properties and static mechanical properties of the composite were analyzed using a dynamic mechanical analyzer, paint film impactor, rubber tensile tester, and other instruments. According to various data, it was verified that APP and EG have a good synergistic flame-retardant effect, and the best proportion of APP and EG is determined to make SAE composite have the best flame retardant, thus achieving the balance between flame-retardant performance and mechanical properties of APP/EG/SAE composite.

## 2. Materials and Methods

### 2.1. Materials

Styrene-acrylic emulsion was purchased from Hengguang New Materials Technology Co., Ltd., Dongguan, China. Expandable graphite (EG) with expansion rate > 300 mL/g (E300) was purchased from Qingdao Yanhai Carbon Materials Co., Ltd., Qingdao, China, with 95–99% carbon content. Mica powder (400 mesh) was purchased from Anhui Chuzhou Wanqiao Silk Mica Powder Factory, Chuzhou, China. Isopropyl tris(dioctyl)titanate pyrophosphate (NDZ-201) was purchased from Wengjiang Chemical Reagent Co., Ltd., Shaoguan, China. Absolute ethanol was purchased from Shanghai Wokai Biotechnology Co., Ltd., Shanghai, China.

### 2.2. Preparation of Modified Ammonium Polyphosphate

A total of 1.5 g of titanate coupling agent (NDZ-20) and 2 g of absolute ethanol were weighed using an electronic balance and added to a 5 mL flat bottom test tube for through mixing and dispersion. The mass ratio of zirconia balls and APP was controlled to be 10:1, and materials were added to the grinding jar at 300 rpm for 10 h wet grinding, during which the mixture of titanate coupling agent and absolute ethanol was added to the grinding jar in 3 parts. After the completion of ball milling, vacuum filtration was performed; the result was rinsed 3 times with absolute ethanol to eliminate residues of physical absorption on the surface of MAPP and dried in a drying oven at 85 °C for 12 h.

### 2.3. Preparation of APP/EG/SAE Composites

**Figure 1:**
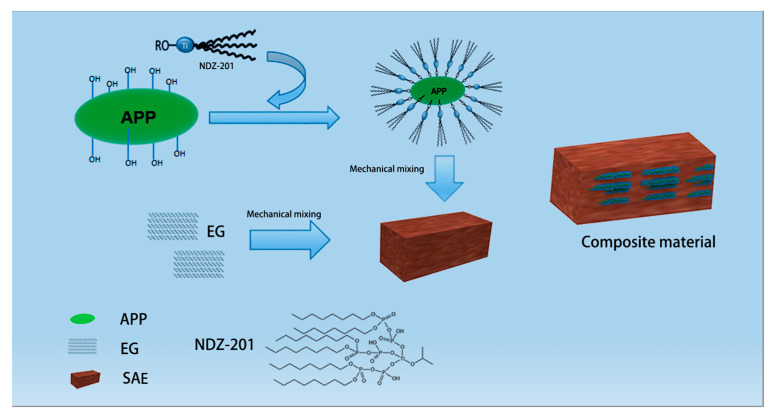
Preparation of APP/EG/SAE composite materials.

Mica powder with a mass fraction of 50% and styrene-acrylic emulsion with a mass fraction of 40% were weighed with an electronic balance and added to a plastic jar; the result was fully stirred and dispersed at 1000 r/min, and recorded as AE-1. Then the modified ammonium polyphosphate (MAPP) and expandable graphite (EG) were added to the styrene-acrylic emulsion instead of the mica powder with a mass fraction of 25% for full stirring, and recorded as AE-2 and AE-3, respectively. The preparation process of composite materials and the principle of NDZ-201 modified APP are shown in Figure 1. the damping fireproof composite materials were produced by replacing the 25% mass fraction of mica powder with MAPP and EG in the same way in different mass ratios (4:1, 3:2, 2:3, 1:4), recorded as AE-4, AE-5, AE-6, and AE-7, respectively.

### 2.4. Characterization

X-ray diffractograms of MAPP powders were obtained by Cu Kα radiation using a DX-2700BH X-ray diffractometer. Surface morphology and fracture analysis of the material microstructure was performed by secondary electron or backscattered electron signals using a VeriosG4 UC type field emission electron microscope, and the sample elements were tested semi-quantitatively using an energy spectrometer. Fourier infrared spectra (FTIR) of MAPP powders were obtained with a T27 spectrometer (BRUKER, Mannheim, Germany) in the wave number range of 500–4000 cm^−1^ obtained by collecting this sample on a KBr sheet. The sessile drop method was adopted to test the contact angle of APP and MAPP based on the static contact angle testing technique of the optical method. Initially, the APP and MAPP were pressed into thin sheets using a press molding machine under pressure of 10 MPa into a circular mold with an inner diameter of 13 mm. Then, using 5 μL water, prepared APP and MAPP sheets were tested on a contact angle measuring instrument (DSA100, Hamburg, Germany). The surface morphology of MAPP particles and composite samples after combustion was observed with a scanning microscope (SEM) of type S-400 at an accelerating voltage of 10 KV. The limiting oxygen index (LOI) test was performed on the composite with a sample size of 80 × 10 × 4 mm^3^ using a limiting oxygen index tester model 5801A according to ASTM D2863. The UL-94 horizontal combustion test was performed by processing the sample size to 125 × 13 × 13 mm^3^ and then testing it with a FTT0082combustion tester. Dynamic mechanical properties analysis (DMA) was performed with a NetzschDMA242E dynamic mechanical analyzer in tensile mode after processing the samples to a size of 30 × 5 × 2 mm^3^. According to GB/T 1732 2020, a 65 mm × 150 mm × 0.5 mm tinplate is used as the test panel, polished with sandpaper, and rinsed with ethanol before drying. The prepared damping coating is applied evenly on the sheet (with a coating thickness of 20 mm) and dried under natural conditions, and the impact resistance of the coating is tested using a paint film impactor (QCJ-50). According to the GB/T 5210 2006 standard, the adhesion magnitude between paint and tinplate was tested with an AI-7000-SU2 universal testing machine. According to the GB/T 528 2009 standard, the sample size is 75 mm long, the parallel section width is 5 mm, the test span is 40 mm, and the tensile rate is 1 mm/min. The tensile strength of the sample was tested using an AI-3000 rubber tensile tester produced by Taiwan Hi-Tech Testing Instruments Co. (Kaohsiung City, Taiwan).

## 3. Results and Discussion

### 3.1. X-ray Diffraction Analysis

**Figure 2:**
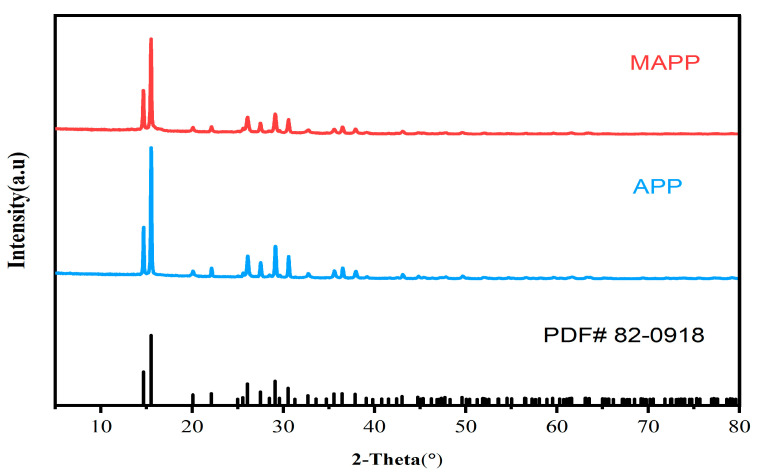
XRD diffraction spectra of APP before and after modification.

In order to study the effect of titanate coupling agent NDZ-201 ball milling modification on the crystal structure of APP, XRD tests were conducted on APP before and after modification, and the test results are shown in Figure 2. The XRD diffraction pattern of APP was compared with the standard PDF#82-0918 pattern, and it was found that the two basically matched, which proved that the crystal type of APP was APP-II. The positions of diffraction characteristic peaks of APP after modification (2θ = 14.6°, 15.4°, 26°, 27.4°) did not change. In addition, no new diffraction peaks appeared in the XRD pattern of MAPP, which indicates that the crystal structure of APP-II was not destroyed after modification by ball milling with titanate coupling agent NDZ-201.

### 3.2. Scanning Electron Microscopy Energy Spectrum Analysis

**Figure 3:**
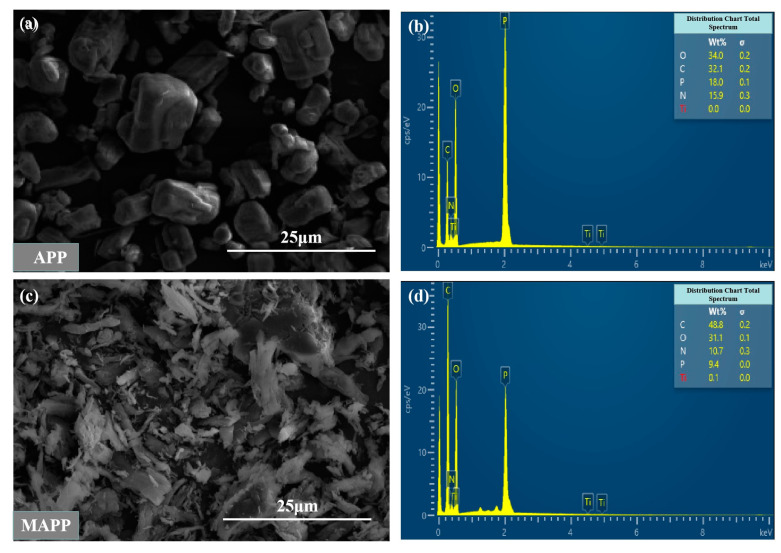
SEM images and corresponding EDS results of samples before and after modification. (**a**) SEM image of APP; (**b**) SEM image of MAPP; (**c**) EDS results of the APP; (**d**) EDS results of the MAPP.

Scanning electron microscopy combined with energy spectroscopy can identify the types and contents of the elements present on the surface of the sample by scanning an electron beam on the surface of the sample. As shown in Figure 3a,c, it can be seen that the APP particles have a regular and neat block structure. After modification, most of the MAPP particles become irregular in shape and their particle sizes decrease, which will potentially increase their dispersibility in MAPP polymers and improve their mechanical and flame-retardant properties [40]. As shown in Figure 3b,d, there are significant differences in surface atomic composition and content between APP and MAPP. The atomic contents of C, O, N, P, and Ti on the surface of APP were 32.1%, 34%, 15.9%, 18%, and 0%, respectively. However, after the sample was modified by coupling agent NDZ-201 ball milling, the atomic contents of C, O, N, P, and Ti on the surface of MAPP were 48.8%, 31.1%, 10.7%, 9.4%, and 0.1%, respectively. Compared to the APP, the carbon and titanium atom contents on the surface of MAPP increased by 16.7% and 0.1%, respectively. The appearance of more titanium and carbon atoms confirms the existence of the MAPP surface titanate coupling agent NDZ-201.

### 3.3. Fourier Transform Infrared Analysis

**Figure 4:**
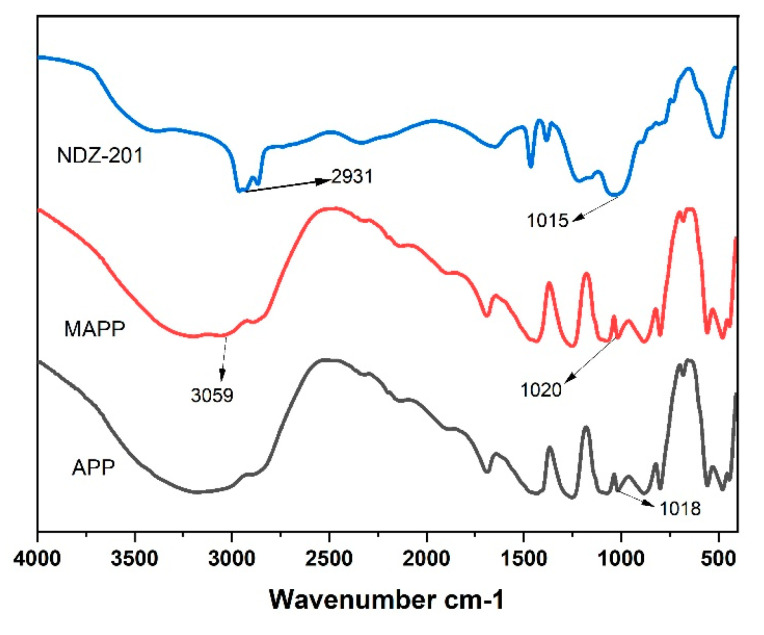
FTIR spectra of NDZ-201, APP, and MAPP.

Figure 4 shows the infrared spectra of APP and modified MAPP. The main characteristic absorption peaks of APP are located at 3177 cm^−1^ (N-H stretching vibration), 1435 cm^−1^ (N-H bending vibration), 1251 cm^−1^ (P=O stretching vibration), 1072 cm^−1^ (P-O symmetric stretching vibration), and 877 cm^−1^ (P-O antisymmetric stretching vibration) [19,41]. The titanate coupling agent NDZ-201 exhibits an antisymmetric vibration peak of C-H near 2934 cm^−1^ [42], and a characteristic absorption peak of Ti-O at 1015 cm^−1^ [43]. When APP was treated with NDZ-201 surface treatment, MAPP showed an antisymmetric vibration absorption peak of C-H at 3059 cm^−1^, showing a blue shift, which may be related to the inductive effect [43]. In addition, MAPP did not exhibit a new characteristic absorption peak at 1020 cm^−1^, which may be due to the overlap between the P-O absorption peak of MAPP at 1072 cm^−1^ and the Ti-O absorption peak of NDZ-201.The appearance of the C-H anti symmetric vibration absorption peak on the surface of MAPP proves that APP was successfully modified by the titanate coupling agent NDZ-201.

### 3.4. Scanning Microscope Analysis

**Figure 5:**
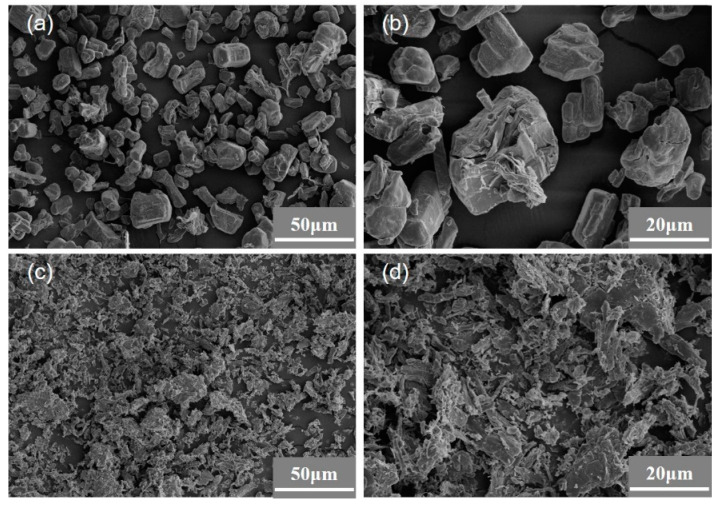
Scanning electron micrograph of APP and MAPP. (**a**) APP magnification ×500; (**b**) APP magnification ×1500; (**c**) MAPP magnification ×500; (**d**) MAPP magnification ×1500.

SEM can be used to observe the surface morphology of APP and MAPP particles. As shown in Figure 5a,b, although the surface of APP particles is smooth, there are some attachments on the surface of some particles, which may be due to the strong hygroscopicity of unmodified APP and partial dissolution on the surface [19]. As shown in Figure 5c,d, after coupling agent NDZ-201 and ball milling modification, the size and morphology of MAPP particles showed significant changes compared to the unmodified APP. The size of MAPP particles decreased and the overall morphology showed a flattening, and a larger surface area was obtained. This may be beneficial for sufficient contact between MAPP and polymer materials, thereby achieving better flame retardancy [44].

### 3.5. Contact Angle Analysis

**Figure 6:**
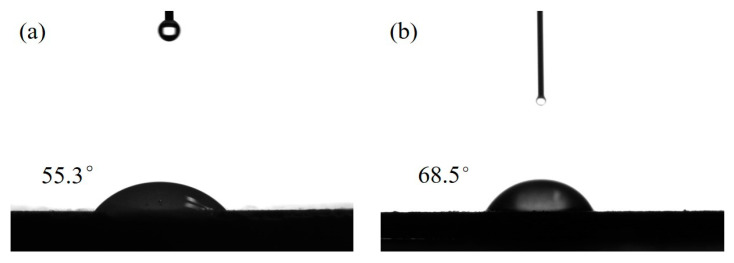
Contact angle image and angle of APP before and after modification ((**a**), APP; (**b**), MAPP).

Figure 6 shows the contact angle before and after modification of APP. Because APP itself contains the hydrophilic groups -OH and -NH_4_^+^ [45], which show strong polarity, the contact angle measured by the goniometric method is small at 55.3°, and the contact angle of MAPP modified by coupling agent is 68.5°. Compared to the modified MAPP powder, the contact angle increased, which indicates that NDZ-201 coupling agent successfully coated the surface of APP.

### 3.6. Compatibility Analysis

**Figure 7:**
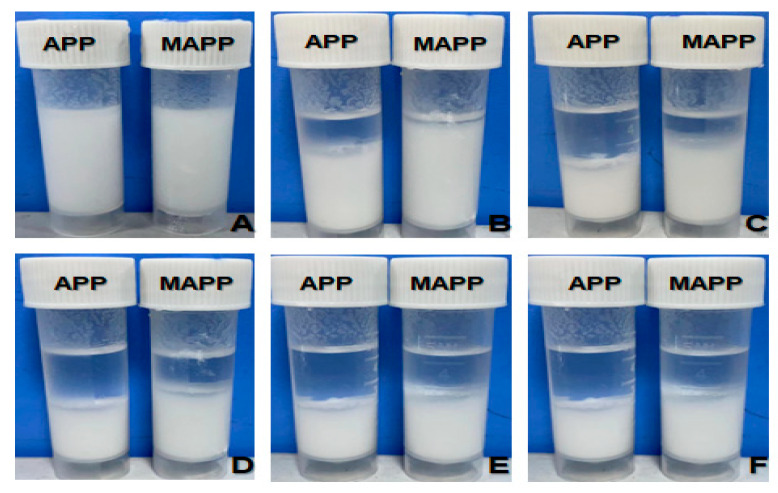
Settlement behavior of APP/MAPP powder in liquid paraffin at different times after ultrasound ((**A**): 0 h, (**B**): 0.5 h, (**C**): 3 h, (**D**): 12 h, (**E**): 48 h, (**F**): 72 h).

Figure 7 shows the settling behaviors of APP and modified NAPPMAPP in liquid paraffin at different times. 0.3 g of each APP and NAPPMAPP were added to the corresponding test tubes containing 4.2 mL of liquid paraffin and sonicated for 30 min and removed and rested. After ultrasonic stirring, the APP began to settle rapidly and reached a stable state after 3 h. However, in comparison, the settling speed of MAPP is relatively slow, reaching a stable state after 48 h, and the final dispersion effect is better than that of APP. Part of the reason for this situation is that the particle size of MAPP obtained after ball milling decreases, resulting in a slower settling speed. Another reason may be that through the NDZ-201 modified APP, organic groups enter the surface of MAPP, thereby improving its hydrophobicity.

### 3.7. Flame Retardancy of APP/EG Composites

**Figure 8:**
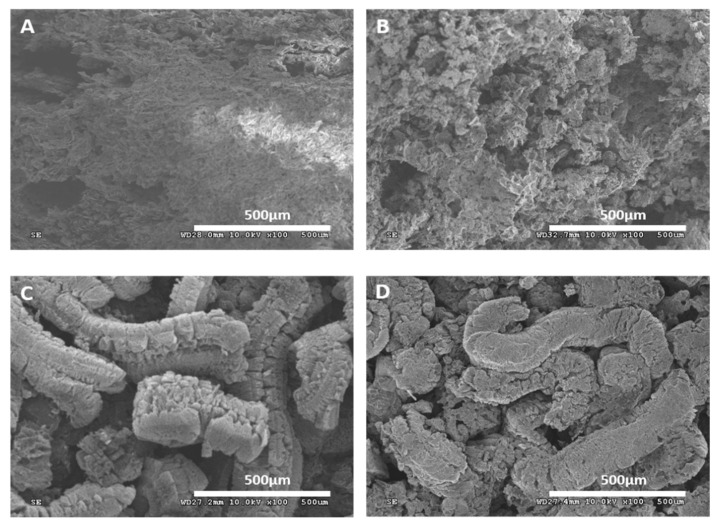
SEM image of MAPP/EG/SAE composite carbon slag obtained after LOI test (**A**): Mica/SAE, (**B**): MAPP/SAE, (**C**): EG/SAE, (**D**): MAPP/EG/SAE.

LOI and UL-94 testing are the most commonly used and effective methods for characterizing the flame retardancy of polymer materials [46,47]. The formula, LOI, and UL-94 test results of flame-retardant SAE composite materials are summarized in Table 1. The LOI value of composite materials containing 50% mass fraction mica powder is very low, at 21.7%, and there is no UL-94 grade. According to the LOI classification, materials with LOI < 22% are flammable materials [14]. When the intumescent flame retardant is added separately, the LOI value of AE-3 composite material is higher than that of AE-2, indicating that EG has a better flame-retardant effect than MAPP in SAE composite material. When MAPP and EG are added together into SAE composite materials, the LOI value of the corresponding SAE composite material is higher than that of the composite material with individual addition of intumescent flame retardants, indicating that the MAPP/EG flame-retardant system exhibits a synergistic effect in LOI testing of SAE composite materials. This situation can be attributed to the following fact: Figure 8 shows the scanning electron microscope images of the carbon residue of samples after LOI testing and combustion of AE-1, AE-2, AE-3, and AE-4. From Figure 8C,D, it can be seen that the carbon layer of the AE-4 sample is more compact than that of AE-2 and AE-3, and the tight carbon layer makes it more difficult for oxygen and heat to enter the inner layer of the material [48], thereby improving the LOI value of the composite material. When the ratio of APP and EG in SAE composite materials is different, the synergistic flame-retardant effect is also different. When the ratio of MAPP and EG in SAE composite materials is 3:2 and 1:4, the materials have good flame-retardant performance, with LOI values of 54.3% and 52.5%, respectively.

### 3.8. Dynamic Mechanical Properties of MAPP/EG Composites

**Figure 9:**
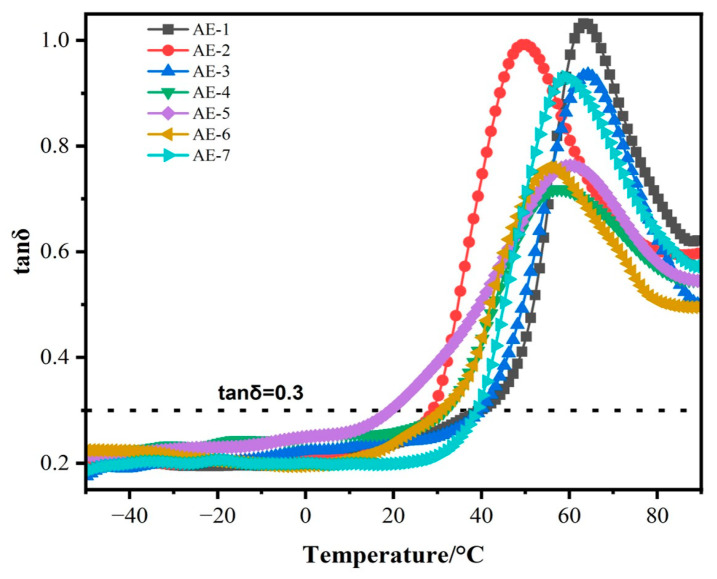
Effect of composite flame retardant on damping performance of composite materials.

**Figure 10:**
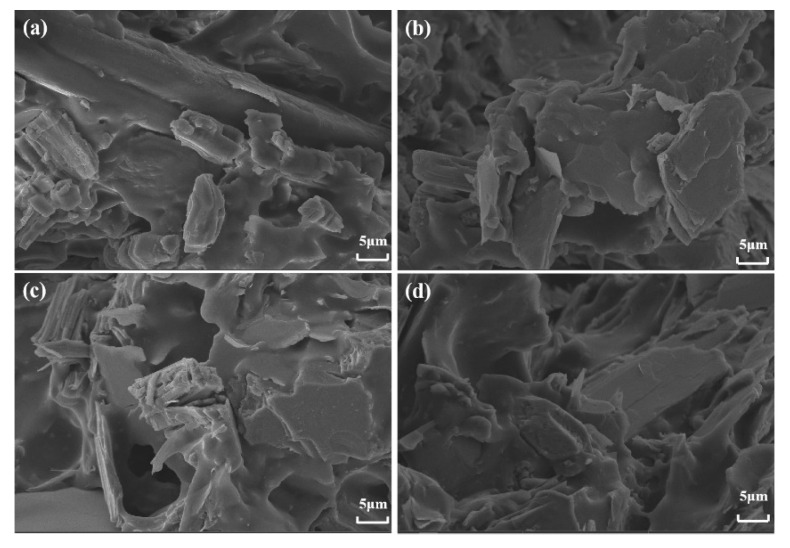
Mechanical cross-sectional morphology of APP/EG/SAE composite materials ((**a**): AE-4, (**b**): AE-5, (**c**): AE-6, (**d**), AE-7).

The loss tangent, tan δ, defined by the ratio E″/E′, is often used to characterize the damping properties of materials [1]. The temperature range of tan δ > 0.3 serves as the standard for evaluating damping materials. In order to study the effect of flame-retardant MAPP/EG on the damping performance of composite materials, the tan δ of the composite material was tested at a given frequency. The relationship with temperature is shown in Figure 9. When intumescent flame retardants are added to SAE in place of some mica according to the formula, the tan δ values of the composites all decreased, especially in the APP/EG composites, where tan δ appeared to decrease with the decrease in EG content. This is because the damping properties of polymer-based damping materials are mainly contributed by the inherent viscoelasticity of the matrix [49], but also by the particle–particle friction and particle–polymer friction when the particles are filled with polymer [2]. As shown in Figure 10d, when the EG content in the composite is the highest, the area where EG is wrapped by the resin is the largest. This results in higher tan δ values due to increased interfacial friction between EG and the polymer. When the ratio of MAPP:EG is 1:4, tan δ of the composite is 0.93 and the damping temperature domain is >56 °C. The composite material has good flame-retardant and damping properties.

### 3.9. Mechanical Properties of MAPP/EG Composites

In order to study the effect of the composite flame-retardant ratio on the mechanical properties of composite materials, adhesion performance, impact resistance performance, and tensile performance of the composite materials were tested. From Table 2, it can be seen that in the composite coating with added flame retardants, when EG accounts for a high proportion of flame retardants, the overall mechanical properties are better. The adhesion of AE-3 and AE-7 is high, at 0.32 MPa and 0.29 MPa, respectively. As shown in Figure 10, the specific surface area of layered EG is large, and can generate more contact area with SAE and enhance the bonding ability of the coating and substrate. When MAPP:EG is 1:4, the impact strength of composite material AE-7 is 50 kg·cm, and the tensile strength is 2.9 MPa.

## 4. Conclusions

In summary, the research shows that the use of titanate coupling agent NDZ-201 for ball milling modification of APP increased the contact angle from 55.3° to 68.5°, improving the hydrophobicity of ammonium polyphosphate and its compatibility with polymers. When MAPP:EG is 1:4 (AE-7), instead of some mica powder, is added to the styrene- acrylic emulsion, it has a synergistic effect on the flame retardancy of the composite. The intermediate products with higher viscosity produced by MAPP can make expandable graphite more compact, isolate heat, and prevent oxygen from entering. At this time, the LOI is 52.5%, which is 141.9% higher than that of AE-1. In addition, when the MAPP/EG mass ratio is 1:4, the composite material also has good dynamic and static mechanical properties. The maximum loss factor of the composite material is 0.93, the damping temperature range is >56 °C, the adhesion is 0.29 MPa, the impact resistance is 50 kg·cm, and the tensile strength is 2.9 MPa. The flame-retardant performance of materials was significantly improved while their damping performance was basically stable, laying a good foundation for the industrial application of new materials.

## Figures and Tables

**Table 1:** LOI and UL-94 results of composite materials.

Sample	Formulation (wt%)			LOI (%)	UL-94 Test	
	Mica Powder	MAPP	EG		Flaming Dripping	UL-94 Rating
AE-1	50	/	/	21.7	Yes	No rating
AE-2	25	25	/	31.2	NO	V-0
AE-3	25	/	25	44.5	NO	V-0
AE-4	25	20	5	45.7	NO	V-0
AE-5	25	15	10	54.3	NO	V-0
AE-6	25	10	15	50.7	NO	V-0
AE-7	25	5	20	52.5	NO	V-0

AE-1: Mica/SAE, AE-2: MAPP/SAE, AE-3: EG/SAE, AE-4: MAPP 20%/EG 5%/SAE, AE-5: MAPP 15%/EG 10%/SAE, AE-6: MAPP 10%/EG 15%/SAE, AE-7: MAPP 5%/EG 20%/SAE.

**Table 2:** Adhesion, impact resistance, and tensile strength of composites.

Samples	Adhesion Strength/MPa	Impact Strength/kg·cm	Tensile Strength/MPa
AE-1	0.35	50	5.75
AE-2	0.23	35	0.88
AE-3	0.32	50	2.07
AE-4	0.14	40	3.24
AE-5	0.15	45	3.27
AE-6	0.27	50	3.05
AE-7	0.29	50	2.9

AE-1: Mica/SAE, AE-2: MAPP/SAE, AE-3: EG/SAE, AE-4: MAPP 20%/EG 5%/SAE, AE-5: MAPP 15%/EG 10%/SAE, AE-6: MAPP 10%/EG 15%/SAE, AE-7: MAPP 5%/EG 20%/SAE.

## Data Availability

The data presented in this study are available on request from the corresponding author.
